# someOne

**DOI:** 10.1007/s10912-024-09894-6

**Published:** 2024-09-16

**Authors:** Martha Gail Rice Skogen

**Affiliations:** Department of Neuromedicine and Movement Science, Faculty of Medicine and Health, Norwegian University of Science and Technology (NTNU), Trondheim, Norway

When the fabric of reality calls to reveal

All that is hidden barely underneath,

We gaze upon something of One who was—

Draped in creased, sterile, covering

Ablaze under the too-blue, shocking lights:

A piercing stillness,

Waves of anxious, tense jitters

All jockeying for our precious attention.

There is no contest:

That face—

Those eyes

Closed for what we call “all time”

Yet we see

How we are changed in one instant ~ 

“Before” was millennia ago.

The sheet covers only the flesh,

Never the soul.

With trembling knife poised,

We too open…

Into this moment of gentle, profound awareness

(No matter how many times it has been done before.)

Time slows into a single message:

To hear—to feel—to breathe—to be here.

We embark upon their story,

The essence of *someOne*

With life laid out before us, we know so little,

Only what they left behind, under this sheet.

Their greatest knowledge

Pours into our reflections of eternity,

We suddenly, finally, let ourselves understand

That the sums of all of us

Melt into one

In this sharing of mutual, deep time,

We trust and honor you:

The *someOne* that you were—and most certainly still are,

For you are the master

In what it means to be alive

Right here, just now, while being so different

On each side of the veil—yet together,

We treasure

Your guidance,

Your wisdom in teaching us that at some point,

We are all

*someOne*

…to *Someone*

